# Reports on Life Assurance

**Published:** 1874-04

**Authors:** 


### Reports on Life Assurance.

Our first report, issued last month, laid down the doctrine that the "labourer is worthy of his hire" in the Department of Life Assurance as well as in other branches of the medical art. We are almost surprised to find that this has been challenged, though in rather an unusual mode. An esteemed correspondent of *The Doctor* having drawn the attention of the actuary of a large company to our reports, has received from him a letter deprecating the view we have adopted. As it is desirable the exact defence of the non-paying offices last put forward should be understood, we quote the words addressed by this actuary to our correspondent. He writes thus:—

"The Life Assurance Department of *The Doctor* begins very badly for assurers and assurance companies, by laying it down as a rule for the guidance of the medical profession that its members are never to fill up a form for an assurance company without a fee. Now, this is an erroneous doctrine, for it very often happens that proposers, in giving reference as to health and habits to a *private friend* name a medical man, and he in some cases won't fill up a *private friend's* report form without a fee; the result is annoyance to the proposer and the office, delay to both, and probably an ill-feeling between all the parties; for how can the doctor expect to be friends after with a gentleman for whom he refused to do such a slight service? or, how can he expect any business for which he would be entitled to payment from from the office he treated so unreasonably? Any man who can read and write can fill up a *friend's* report. It is not necessary that he should be a doctor or belong to any profession, and therefore, when a doctor is asked to act for a friend in the *capacity of a friend*, I don't see that he has any right to demand a fee simply because an insurance company is in question. He might as well ask a fee for acting as reference for a friend taking a house or a farm, or entering into any engagement in which a reference would be necessary.

"If you think well of enlightening the profession on this matter, you may do some of them *a service*. I fear *The Doctor's* doctrine is calculated rather to increase the evil I refer to."

Now here, be it observed, the position that a *medical* report ought not to be paid for is completely abandoned, and we are told that such a friend's report, as any one who can read and write could fill up is sometimes refused without a fee. Most medical men know the difference between a medical report and a friend's reference. Where the applicant has a right to give such reference as he would in taking a house, we can understand a doctor replying without a fee. No medical report can honestly be filled up without an examination of the patient at the time, and every office expects such examination to be carefully made, and all the numerous questions honestly answered. A friend's report is altogether different, though it is scarcely such a reference as would be required in letting a house, for it usually asks about the person's habits and apparent health. Of course, only an unskilled reply is expected, and if we consented to fill up such a form we should never think of examining the patient or asking him a question likely to elicit anything prejudicial to the applicant. We might thus fill up a certificate for a person we had never professionally attended or about whose health we had heard nothing. It is altogether different if the office applies to the doctor of the applicant, because he has already professional knowledge obtained by his attendance, and it is doubtful whether such knowledge should be sought. Any company thus attempting to obtain unfair advantage runs such a risk of getting a number of bad lives that we would recommend all who dersired to insure to avoid that office, for it is the interest of those insured that the company should be stable.—*The Doctor.*

